# Thinking beyond *Opisthorchis viverrini* for risk of cholangiocarcinoma in the lower Mekong region: a systematic review and meta-analysis

**DOI:** 10.1186/s40249-018-0434-3

**Published:** 2018-05-17

**Authors:** Jennifer A. Steele, Carsten H. Richter, Pierre Echaubard, Parichat Saenna, Virginia Stout, Paiboon Sithithaworn, Bruce A. Wilcox

**Affiliations:** 1 Department of Infectious Disease and Global Health, Tufts University, Cummings School of Veterinary Medicine, North Grafton, MA USA; 2 Global Health Asia, Integrative Education and Research Programme, Faculty of Public Health, Faculty of Public Health Studies, Bangkok, Thailand; 3 Center for Mountain Ecosystem Studies, Kunming Institute of Botany, Chinese Academy of Sciences, Kunming, China; 4 Department of Biology, Laurentian University, Greater Sudbury, ON Canada; 5 Department of Science Education, Khon Kaen University, Faculty of Education, Khon Kaen, Thailand; 6 Department of Parasitology, Khon Kaen University, Faculty of Medicine, Khon Kaen, Thailand

**Keywords:** Cholangiocarcinoma, Risk factors, *Opisthorchis viverrini*, Mekong, Southeast Asia, Thailand, Public health

## Abstract

**Background:**

Cholangiocarcinoma (CCA) is a fatal bile duct cancer associated with infection by the liver fluke, *Opisthorchis viverrini*, in the lower Mekong region. Numerous public health interventions have focused on reducing exposure to *O. viverrini*, but incidence of CCA in the region remains high. While this may indicate the inefficacy of public health interventions due to complex social and cultural factors, it may further indicate other risk factors or interactions with the parasite are important in pathogenesis of CCA. This systematic review aims to provide a comprehensive analysis of described risk factors for CCA in addition to *O. viverrini* to guide future integrative interventions.

**Main body:**

We searched five international and seven Thai research databases to identify studies relevant to risk factors for CCA in the lower Mekong region. Selected studies were assessed for risk of bias and quality in terms of study design, population, CCA diagnostic methods, and statistical methods. The final 18 included studies reported numerous risk factors which were grouped into behaviors, socioeconomics, diet, genetics, gender, immune response, other infections, and treatment for *O. viverrini*. Seventeen risk factors were reported by two or more studies and were assessed with random effects models during meta-analysis. This meta-analysis indicates that the combination of alcohol and smoking (*OR* = 11.1, 95% *CI*: 5.63–21.92, *P* <  0.0001) is most significantly associated with increased risk for CCA and is an even greater risk factor than *O. viverrini* exposure. This analysis also suggests that family history of cancer, consumption of raw cyprinoid fish, consumption of high nitrate foods, and praziquantel treatment are associated with significantly increased risk. These risk factors may have complex relationships with the host, parasite, or pathogenesis of CCA, and many of these risk factors were found to interact with each other in one or more studies.

**Conclusions:**

Our findings suggest that a complex variety of risk factors in addition to *O. viverrini* infection should be addressed in future public health interventions to reduce CCA in affected regions. In particular, smoking and alcohol use, dietary patterns, and socioeconomic factors should be considered when developing intervention programs to reduce CCA.

**Electronic supplementary material:**

The online version of this article (10.1186/s40249-018-0434-3) contains supplementary material, which is available to authorized users.

## Multilingual abstracts

Please see Additional file 1 for translations of the abstract into the six official working languages of the United Nations.

## Background

Cholangiocarcinoma (CCA) is a malignant tumor of the biliary tract occurring in high incidence in the lower Mekong region, including Thailand, Laos, Cambodia, and Vietnam. Annual CCA incidence in these areas ranges from 93.8 to 317.6 per 100 000 people, with most cases being fatal within 1 to 2 years of diagnosis [1–4]. CCA in this region is associated with infection by the Southeast Asian liver fluke, *O. viverrini* [5, 6] which is endemic in this area. *O. viverrini* is a foodborne helminth spread by ingestion of infected raw or undercooked cyprinoid fish in traditional local dishes [5]. *O. viverrini* infection prevalence is up to 70% in some areas, with estimates of up to 10 million human infections in the lower Mekong region [6–9]. *O. viverrini* has been classified as a group 1 carcinogen in humans by the International Agency for Research on Cancer (IARC) since 1994 [8, 10].

Chronic infection with *O. viverrini* may contribute to CCA development through induction of host immune response and inflammation in the bile ducts over the course of decades [5, 8, 11]. Many interventions trying to reduce CCA incidence have focused on reducing *O. viverrini* infection in humans by incentivizing behavioral modification to reduce consumption of traditional raw fish dishes and reduce defecation in rice fields [12, 13]. However, prevalence of *O. viverrini* infection in the lower Mekong region still remains high [14]. Challenges to reducing CCA incidence have been associated with the complexity of CCA etiology, difficulty of changing traditional cultural practices, and risk perception among the population [15]. Recent publications recognizing these challenges have advocated for integrated approaches to reduce *O. viverrini* infection and CCA incidence in the Mekong region [12, 13, 16].

*O. viverrini* infection has long been believed to be the primary risk factor, but numerous studies have also focused attention on other risk factors for CCA independently or in conjunction with *O. viverrini* [17, 18]. For instance, men develop CCA at up to twice the rate as women, but the difference in prevalence of *O. viverrini* infection between women and men does not match this difference [3, 18], providing one indication that other risk factors may be impacting men more than women. In addition, the quantitative correlation between *O. viverrini* infection prevalence and CCA incidence in Thailand is not consistent for all regions and may indicate importance of other risk factors [5]. An examination of other risk factors for CCA and current understanding of CCA pathogenesis is warranted in this regard. Inclusion of ecological perspectives to frame future research and provide new approaches to the problem of CCA has been proposed to overcome the limited progress on CCA reduction [1, 19]. Past research has explored many other risk factors, and this systematic review and meta-analysis aims to compile individual results and quantify the most important risk factors and their relationships to development of CCA in the lower Mekong region. Here we provide the first comprehensive review and meta-analysis of the body of research on CCA risk with quantitative analysis of risk factors described so far.

## Methods

### Search strategy and selection criteria

Following Preferred Reporting Items for Systematic Reviews and Meta-Analyses (PRISMA) guidelines [20], five international databases were searched on February 8, 2015: MEDLINE, SCOPUS, Web of Science, The Cochrane Library, and Science Direct. Citations of relevant references were considered to identify any further references missed by the database queries. Thai language publications in local journals or repositories were identified as the most important source of grey literature, so the Thai National Cancer Institute records, Khon Kaen University Research Journal, Thai Cancer Journal, Srinagarind Hospital Cancer Unit reports, Srinagarind Medical Journal, Thai Bureau of Epidemiology reports, and Ubon Ratchathani University Journal of Science and Technnology were searched in Thai to identify additional references. The most inclusive search terms (“cholangiocarcinoma” AND “opisthorchis”) were used for all queries, and references published at any time and in any language were considered for review.

The titles and abstracts of all references were screened by at least two reviewers for relevance to identify studies that reported primary research involving risk of CCA in humans in the *O. viverrini* endemic lower Mekong region. All references matching these basic criteria were evaluated in full text for inclusion in the final systematic review, based on fulfillment of all of the following a priori inclusion criteria: the study design includes 1) human patients in the *O. viverrini* endemic lower Mekong region, 2) CCA diagnosis, 3) a comparison group without CCA, and 4) examination of risk factors for CCA in addition to *O. viverrini*. Included references were evaluated to determine weaknesses in study design in terms of limitations, risk of bias, choice of study population, definition of CCA cases, matching of controls, sample size, and statistical methods to assess the overall quality of the final set of references.

### Data extraction and coding

At least two reviewers evaluated the full text of each included reference and extracted data for factors that were reported to increase, decrease, or have no significant effect on risk of CCA. Case and control exposure data or odds ratio and 95% confidence interval (*CI*) were recorded for risk factors reported in two or more studies for meta-analysis. Characteristics of each study including selection of the study population, diagnostics, study design, consideration of confounding, sample size, and statistical methods were extracted to examine sources of bias and heterogeneity. If more than one control group was included in a study, data from the healthy control group was used for consistency across studies. During data extraction, if overlapping datasets from the same patients were encountered in multiple included studies, data from the most complete report was included to avoid duplication of patient data in meta-analysis.

### Statistical analysis

Random effects models of log odds ratios were used to estimate summary measures for risk factors reported comparably by two or more studies. Random effects models were chosen to account for heterogeneity across included studies since heterogeneity was expected due to factors such as differences in patient source, diagnosis of CCA, measurement of exposures, and sample size. The I^2^ statistic was used to assess the degree of heterogeneity across studies included in each meta-analysis. R (version 3.3.2, metafor package) was used for statistical analyses [21].

## Results

**Fig. 1:**
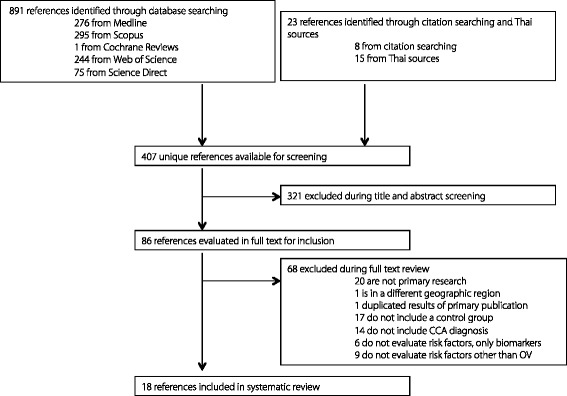
PRISMA search strategy summary

Queries returned 390 unique references from the five scientific databases and 15 from Thai language sources. Of these, 78 were identified as potentially relevant. Citation searching within relevant references and retrieved reviews identified eight additional references as potentially relevant. Of the 86 potentially relevant references, 18 met all inclusion criteria and were included in this systematic review (Fig. 1). A wide range of risk factors for CCA were reported, which were grouped into the following categories: behaviors, socioeconomics, diet, genetics, immune response (including anti-*O. viverrini* antibody response), gender, other infections, or treatment for *O. viverrini* infection (Table 1).

**Table 1:** Summary of included references evaluating risk factors for cholangiocarcinoma

Reference	Year	Country	Source of patients or samples	Study design	Sample size: cases/controls	Risk factor categories reported	Measure of effect
Parkin et al. [22]	1991	Thailand	Maharat Nakorn Ratchasima Hospital, Ubon Ratchathani Hospital, National Cancer Institute	case-control	103/103	behaviors, socioeconomics, diet, immune response, other infection	odds ratio
Itoh et al. [35]	1994	Thailand/ Japan	Khon Kaen province hospitals, Japan	case-control	47/68	immune response, gender	mean or proportion difference
Haswell-Elkins et al. [46]	1994	Thailand	Villages in Khon Kaen and Mahasarkham provinces	cross-sectional survey	15 CCA cases of 1807 patients screened	gender	odds ratio
Honjo et al. [23]	2005	Thailand	Nakhon Phanom province hospitals	case-control	129/129	behaviors, socioeconomics, diet, genetics, treatment	odds ratio
Jinawath et al. [31]	2006	Thailand/ Japan	Srinagarind Hospital and Kyoto University Hospital	case-control	20/10	genetics	median gene expression
Marahatta et al. [55]	2006	Thailand	LFCRC Repository^a^	case-control	30/30	genetics	odds ratio
Prawan et al. [29]	2005	Thailand	Srinagarind Hospital	case-control	216/233	genetics	odds ratio
Poomphakwaen et al. [2]	2009	Thailand	Khon Kaen Cohort Study	nested case-control	108/108	behaviors, diet, treatment, genetics	odds ratio
Srivatanakul et al. [38]	2010	Thailand	Nakhon Phanom province hospitals	case-control	106/106	other infections, immune response	odds ratio
Songserm et al. [27]	2011	Thailand	Khon Kaen Cohort Study	nested case-control	219/438	genetics, diet, socioeconomics	odds ratio
Sripa et al. [37]	2012	Thailand	Khon Kaen area villages, non-OV endemic Thailand, LFCRC Repository^a^	case-control	121/21	immune response	odds ratio
Yongvanit et al. [32]	2012	Thailand	Khon Kaen province hospitals	case-control	13/10	genetics	mean difference
Songserm et al. [24]	2012	Thailand	Khon Kaen Cohort Study	nested case-control	219/438	behaviors, socioeconomics, diet, genetics	odds ratio
Zeng et al. [30]	2013	Thailand	Ubon Ratchathani Cancer Center Hospital	case-control	105/105	genetics	odds ratio
Manwong et al. [25]	2013	Thailand	Sappasit Prasong Hospital and Ubon Ratchathani Cancer Center	case-control	123/123	socioeconomics, diet, treatment, behaviors, immune response	odds ratio
Plieskatt et al. [33]	2014	Thailand	LFCRC Repository^a^	case-control	16/13	genetics	mean difference
Songserm et al. [26]	2014	Thailand	Khon Kaen Cohort Study	case-control	219/438	behaviors, genetics	odds ratio
Prayong et al. [34]	2014	Thailand	Srinagarind Hospital and community	case-control	79/80	genetics	odds ratio

^a^Liver Fluke and Cholangiocarcinoma Research Center (LFCRC) Biological Repository, Khon Kaen University, Thailand

### Behaviors

Smoking and alcohol consumption were the two behavioral factors reported in the included references, and six references evaluated smoking and/or alcohol consumption [2, 22–26], of which three examined the interaction between these factors [23, 24, 26]. Only one study reported smoking alone as a significant risk factor [26] with five reporting no significant risk from smoking alone [2, 22–25]. Alcohol consumption was associated with significantly increased risk of CCA by all but one report [22]. One study examining types and frequency of alcohol consumption found that increased frequency or increased units of alcohol consumption per day were associated with an increased risk [2]. Red whiskey was significantly associated with CCA but beer and sato were not [2]. Finally, three studies reported that the combination of smoking and alcohol consumption multiplied risk for CCA development [23, 24, 26].

### Socioeconomics

Two studies reported significantly reduced risk of CCA associated with higher educational level [22, 27] and two reported no significant difference in risk [23, 24]. Further differentiation in one study found that people completing at least primary education exhibited nearly 70% reduction in risk and those with secondary or higher education 80% reduction in risk [27]. Other socioeconomic factors reported included marital status and occupation, neither of which was significantly associated with risk of CCA [23, 27]. Household socioeconomic characteristics evaluated were non-pipe water source, which was associated with increased risk by one study, and toilet location, which was not associated with risk [23]. The socioeconomic factors reported by the included studies represent a range of factors that are not likely directly related to CCA pathogenesis but may have complex relationships with other risk factors.

### Diet

Numerous dietary factors, including local dishes and individual foods or ingredients, were evaluated by seven of the included studies. A summary of foods for which odds ratios were reported is provided in Table 2. The most commonly evaluated dietary factors were dishes containing raw, undercooked, or fermented fish products, since they may transmit *O. viverrini*. While one study found no significant association [22], three studies reported significantly increased risk for CCA associated with consumption of raw fish, common in the traditional diet in Northeast Thailand [23–25], with one study finding increasing risk with increasing frequency of consumption [24]. The consumption of fermented fish dishes was not found to be associated with significant risk [22, 23, 25]. Foods associated with increased risk of developing CCA, besides raw fish products, were fermented meats, sausages, and betel nuts, all of which contain volatile nitrosamines, which are associated with increased risk [28]. Fermented meat products are particularly high in nitrosamines, and increased risk for developing CCA was associated with consumption of volatile nitrosamines in foods [28], fermented pork [23], and fermented beef sausage [24]. Among other fresh or raw meat and seafood dishes, only frequent consumption of raw beef or pork significantly increased risk of CCA [25]. Chewing betel nuts was also commonly evaluated, with one study reporting increased risk [22], but two others reporting no association [23, 25]. Three studies reported significantly reduced risk of developing CCA associated with consumption of fresh fruits and vegetables [2, 22, 24], one of which additionally reported reduced risk associated with milk, salted fish, and rice [22].

**Table 2:** Dietary risk factors and association with CCA

	Food	Odds ratio	95% *CI*	Cases^a^	Comparison	Source
Raw fish dishes						
	koi pla	10.2	3.05–34.1	77	never vs daily	Songserm 2012 [24]
	raw cyprinoid fish	3.4	1.05–11.01	100	no or < 1/month vs 3/week	Manwong 2013 [25]
	raw fish	2.94	1.24–6.96	75	none vs > 2/month	Honjo 2005 [23]
	koi pla	2.5	1.05–5.74	104	never vs weekly	Songserm 2012 [24]
	koi pla	1.6	0.7–3.5	97	< monthly vs more	Parkin 1991 [22]
Fermented fish or meat dishes						
	nitrate containing foods^b^	4.91	1.04–23.24	216	never vs often	Poomphakwaen 2009 [2]
	fermented fish or pork	4.5	1.3–15.54	71	none vs > 2/month	Honjo 2005 [23]
	beef sausage	3.7	1.28–10.7	95	never vs daily	Songserm 2012 [24]
	cooked pla ra	3	0.31–28.84	34	no or < 1/month vs 1–4/week	Manwong 2013 [25]
	pla ra, pla chao	2.25	0.92–5.53	129	< 3/day vs > 3/day	Honjo 2005 [23]
	sour shrimp	1.93	0.47–7.93	101	no or < 1/month vs 3/week	Manwong 2013 [25]
	uncooked pla ra	1.75	0.74–4.19	86	no or < 1/month vs 3/week	Manwong 2013 [25]
	pla ra	1.6	0.9–2.8	96	< 3/daily vs more	Parkin 1991 [22]
	pla chom	1.6	0.57–4.51	94	no or < 1/month vs 3/week	Manwong 2013 [25]
	sour beef or pork	1.37	0.51–3.65	88	no or < 1/month vs 3/week	Manwong 2013 [25]
	Chinese or Northeast sausage	1.3	0.51–3.35	103	none vs > 1/month	Honjo 2005 [23]
	salted fish or meat	1.01	0.41–2.44	113	none vs > 6/year	Honjo 2005 [23]
	pla chom	1	0.5–1.9	98	< monthly vs more	Parkin 1991 [22]
	pla som	0.7	0.4–1.3	97	never vs ever	Parkin 1991 [22]
	salted fish	0.5	0.2–0.9	96	< monthly vs more	Parkin 1991 [22]
	pla chao	0.5	0.2–1.4	98	< monthly vs more	Parkin 1991 [22]
Other fish and seafood						
	total fish and shellfish	1.31	0.56–3.04	147	< 0.4 vs > 0.8/day	Poomphakwaen 2009 [2]
	raw shellfish	0.85	0.27–2.74	97	no or < 1/month vs 3/week	Manwong 2013 [25]
Fresh meat dishes						
	raw beef or pork	3.38	1.25–9.12	91	no or < 1/month vs 3/week	Manwong 2013 [25]
	raw sausage	1.19	0.48–2.97	86	no or < 1/month vs 3/week	Manwong 2013 [25]
	roast meat	0.74	0.25–2.18	78	< 2/week vs > 1/day	Honjo 2005 [23]
	poultry	0.62	0.23–1.7	216	< 0.2 vs > 0.4/day	Poomphakwaen 2009 [2]
	beef and pork	0.31	0.08–1.2	216	< 0.45 vs > 1/day	Poomphakwaen 2009 [2]
Fruits and vegetables						
	fruit	1.72	0.85–3.47	98	no or < 1/month vs 3/week	Manwong 2013 [25]
	fresh fruit	1.63	0.64–4.12	107	< 1/week vs > 2/week	Honjo 2005 [23]
	cooked fresh vegetables	1.48	0.62–3.51	110	< 3/week vs > 1/day	Honjo 2005 [23]
	vegetables	1.36	0.61–3.02	95	no or < 1/month vs 3/week	Manwong 2013 [25]
	fermented vegetables or fruits	1.18	0.44–3.2	86	< 4/week vs > 2/day	Honjo 2005 [23]
	fruit	0.7	0.33–0.98	219	< 52 vs > 52 times/month	Songserm 2012 [24]
	fresh vegetables	0.67	0.27–1.67	95	< 1/week vs > 2/day	Honjo 2005 [23]
	vegetables	0.6	0.27–1.36	190	< 1.7 vs > 1.7/day	Poomphakwaen 2009 [2]
	fresh fruit	0.5	0.3–0.9	93	< 3/month vs more	Parkin 1991 [22]
	fruit	0.44	0.23–0.85	156	< 1 vs > 1/day	Poomphakwaen 2009 [2]
	vegetables	0.4	0.23–0.76	219	< 52 vs > 52/month	Songserm 2012 [24]
Grains						
	sticky rice	2.6	1.4–4.9	94	< 2/week vs more	Parkin 1991 [22]
	rice	1.35	0.55–3.27	99	< 4/year vs > 3/month	Honjo 2005 [23]
	sticky rice	1.03	0.47–2.24	125	< 3/day vs > 3/day	Honjo 2005 [23]
	rice	0.7	0.4–1.2	94	< 3/day vs more	Parkin 1991 [22]
Betel nut						
	betel nut	6.4	1.1–39.3	n/a	< 1/day vs more	Parkin 1991 [22]
	betel nut	3	0.81–11.08	123	yes or no	Manwong 2013 [25]
	betel nut	0.69	0.18–2.7	122	regular vs never chewing	Honjo 2005 [23]
Other						
	somtam	1.9	0.55–6.62	42	never vs daily	Songserm 2012 [24]
	monosodium glutamate	1.8	1.0–3.2	97	< 2/day vs more	Parkin 1991 [22]
	milk	0.5	0.3–0.9	95	< monthly vs more	Parkin 1991 [22]

Foods in each category are organized by odds ratio in descending order

^a^Number of CCA cases included in the comparison

^b^Nitrate containing foods are fermented and salted items that are high in volatile nitrosamines

### Genetics

Many included studies investigated genetic traits, with 12 reporting risk associated with polymorphisms or expression profiles of 18 distinct genes, summarized in Table 3. Three studies reported risk related to family history of cancer, which may be driven by family genetics as well as behavioral, socioeconomic, and environmental exposures shared within families. The earliest study evaluating a family history of cancer found no significant risk [22] however two later studies found significantly increased risk of CCA with family history [2, 25].

**Table 3:** Genetic risk factors for CCA

Gene	Title	Function	CCA Risk	Source
OGG1	8-oxoguanine DNA glycosylase 1	DNA repair	not significant^a^	Zeng 2013 [30]
OGG1	8-oxoguanine DNA glycosylase 1	DNA repair	not significant^a^	Songserm 2014 [26]
PARP-1	poly-ADP-ribose polymerase 1	DNA repair	not significant^a^	Zeng 2013 [30]
XRCC1	X-ray repair cross-compelementing protein 1	DNA repair	not significant^a^	Songserm 2014 [26]
XRCC1	X-ray repair cross-complementing protein 1	DNA repair	not significant^a^	Zeng 2013 [30]
IL-6R	interleukin 6 receptor	inflammation	increased C allele frequency decreased risk	Prayong 2014 [34]
CHST4	sulfotransferase	metabolism	upregulated in Thai CCA	Jinawath 2006 [31]
CYP1A2	cytochrome P450 oxidase	metabolism	CYP1A2*1A/*1A decreased risk in men^a^	Prawan 2005 [29]
CYP2A6	cytochrome P450 oxidase	metabolism	increased expression in CCA	Yongvanit 2012 [32]
CYP2E1	cytochrome P450 oxidase	metabolism	decreased expression in CCA	Yongvanit 2012 [32]
GST01	glutathione-s-transferase	metabolism	GST01*D140 increased risk	Marahatta 2006 [55]
GST02	glutathione-s-transferase	metabolism	not significant	Marahatta 2006 [55]
GSTM1	glutathione-S-transferase	metabolism	not significant^a^	Honjo 2005 [23]
GSTT1	glutathione-S-transferase	metabolism	not significant^a^	Honjo 2005 [23]
MTHFR	methylenetetrahydrofolate reductase	metabolism	A1298C CC variant increased risk^a^	Songserm 2011 [27]
MTHFR	methylenetetrahydrofolate reductase	metabolism	A1298C CC variant increased risk^a^	Songserm 2012 [24]
NAT1	catalyze N and O acetylation	metabolism	NAT1*11 decreased risk	Prawan 2005 [29]
NAT2	catalyze N and O acetylation	metabolism	NAT2*13, *6B, and *7A decreased risk	Prawan 2005 [29]
SULT1C1	sulfotransferase	metabolism	upregulated in Thai CCA	Jinawath 2006 [31]
UGT1A10	UDP-glucuronosyltransferase	metabolism	upregulated in Thai CCA	Jinawath 2006 [31]
UGT2B11	UDP-glucuronosyltransferase	metabolism	upregulated in Thai CCA	Jinawath 2006 [31]

^a^Gene has significant interaction with or modification of other risk factors, see Table 4

Genes regulating metabolic functions were commonly studied as many have been identified as risk factors for various cancers due to alteration of metabolism of environmental carcinogens [29]. Polymorphisms of GSTM1 or GSTT1 alone did not correlate with risk for CCA [23] however combined polymorphisms in DNA repair and glutathione-S-transferase genes [30] were associated with reduced risk of CCA. Reduced expression of growth factor signaling genes [31] was also associated with significantly reduced risk of CCA. The CYP1A2 gene was not associated with overall risk of CCA, but the CYP1A2*1A/*1A polymorphism decreased risk of CCA in males [29]. The NAT1*11 allelle and NAT2*13, *6B, and *7A alleles also significantly decreased risk of CCA [29]. Genes related to xenobiotic and endobiotic metabolism, including UGT1A10, UGT2B11, CHST4, and SULT1C1, were expressed at significantly higher levels in *O. viverrini* associated CCA cases [31]. Expression of the CYP2A6 was increased, and expression of CYP2E1 was decreased in CCA cases [32]. miRNA dysregulation was greatest in moderately differentiated CCA patients [33] however genetic expression and miRNA profile changes are likely a result of carcinogenesis and do not necessarily reflect individual risk factors but may provide information for diagnosis or examination of other interactions among risk factors. One study evaluated polymorphisms in the IL-6 receptor gene and found that increased frequency of the C allele and decreased frequency of the A allele in the 48 892 A/C polymorphism of exon 9 decreased risk of CCA [34]. The DNA repair genes OGG1, PARP-1, and XRCC1 were not found to influence risk of CCA when considered alone but may have interaction with other factors [26, 30].

### Immune response

The immune response to *O. viverrini* and measurement of anti-*O. viverrini* antibodies has long been used to assess past and current *O. viverrini* exposure in patients. In this review, with the primary goal of identifying risk factors other than *O. viverrini* infection, we assessed the nature of the immune response against *O. viverrini* in the context of CCA risk. Five studies reported significantly increased risk of CCA with positive *O. viverrini* antibody titers [22, 23, 25, 35, 36] and several made notable observations about this relationship. Anti-*O. viverrini* antibodies were associated with increased risk of CCA, but *O. viverrini* eggs being shed in the feces were not [22]. This is interesting in that the presence of the parasite alone had no relationship with CCA diagnosis, but an elevated immune response against the parasite was associated with increased risk. While an increased antibody titer can indicate a greater intensity of infection or repeated exposure to the parasite, this could also indicate that differing individual immune responses to *O. viverrini* infection are related to risk of CCA development, possibly through inflammatory responses [5]. IL-6 is a pro-inflammatory cytokine with a suspected role in the pathogenesis of CCA, and patients with the greatest elevation in plasma IL-6 had over 100 times the odds of developing CCA [37].

### Other infections

Chronic viral hepatitis is a common cause of hepatocellular cancers and was investigated as a risk for CCA by two studies. Both found that positive hepatitis B antigen titers alone were not a significant risk [22, 38], but one found that anti-hepatitis C virus titers were associated with significantly increased risk for developing CCA [38]. Furthermore, patients who were positive for hepatitis B antigen and/or hepatitis C antibodies had significantly increased odds of developing CCA over patients who were negative for both viruses [38].

### Treatment

The anthelmintic drug praziquantel is commonly used to treat *O. viverrini* infection, and, since it is a highly effective treatment, it has been used in community mass drug administration campaigns to reduce prevalence of infection [6, 39]. The relationship between *O. viverrini* infection, praziquantel treatment, and CCA has been investigated since animal model studies first suggested a potential increased risk of CCA due repeated *O. viverrini* infection and praziquantel treatment, possibly related to oxidative stress following praziquantel treatment [40–42]. In human epidemiological studies, the association between repeated praziquantel administration and CCA has also been noted [3, 6, 43], and past treatment with praziquantel has also been associated with increased likelihood of subsequent *O. viverrini* infection [39]. However, the exact nature of the relationship between praziquantel treatment and pathogenesis of CCA is not clear, and a previous systematic review found no significant association between praziquantel and CCA [44]. In this review, three included epidemiological studies found that praziquantel was not associated with risk of developing CCA [2, 25, 26], while one study found that it was associated with increased risk [23].

### Gender

The Thai National Cancer Registry reports indicate that CCA affects over twice as many men as women [45]. While most studies included in this review matched cases and controls based on sex and did not evaluate gender as a risk factor, two studies reported increased risk of CCA in men [22, 46]. The exact reasons for the increased risk in men is not yet fully understood, but these studies reported several relevant findings related to gender and risk of developing CCA. Men are known to have increased prevalence of *O. viverrini* infection compared to women, due in part to their social behaviors related to consumption of raw fish dishes, and that they also have greater consumption of high nitrosamine foods, as well as increased smoking and alcohol use [15]. In one study, the percentage of CCA cases with *O. viverrini* infection in men was 72%, compared with 62% in women, suggesting that *O. viverrini* infection may be a greater risk in men [22]. Likewise, men with greater *O. viverrini* infection intensity had increased risk of developing CCA compared to women [46] (Table 4). Female CCA cases had lower *O. viverrini* infection and antibody positivity rates, though no difference in parasite burden or anti-*O. viverrini* antibodies had been observed in community studies of *O. viverrini* prevalence, also raising questions about the increased risk of developing CCA in men with *O. viverrini* infection and other risk factors in women [35].

**Table 4:** Significant relationships between risk factors for CCA

	Risk Factors
Increased risk of CCA	alcohol x smoking [23, 26] alcohol x fermented fish [23] GSTM1 x *O. viverrini* antibody [23] GSTT1 x alcohol [23] MTHFR x raw fish [24, 27] MTHFR x fermented or processed meat [24] MTHFR x *O. viverrini* antibody [27] male x *O. viverrini* antibody [22, 46] XRCC1 x OGG1^a^ [26] XRCC1 x smoking [26] OGG1 x smoking [26]
Decreased risk of CCA	CYP1A2 x male [29] CYP1A2 x smoking [29] GSTM1 x OGG1 [30] XRCC1 x OGG1^a^ [26]

Individual gene risk information in Table 3

^a^XRCC1 and OGG1 genes both exhibit multiple polymorphisms, and depending on the combination of polymorphisms present in the individual, the interaction between XRCC1 and OGG1 may increase or decrease risk

### Relationships between risk factors

In addition to the interaction of the effects of smoking and alcohol consumption mentioned above, eight studies evaluated other relationships between risk factors, summarized in Table 4. Drinkers who ate increasing amounts of the fermented fish dishes were at increased risk of developing CCA [23]. Alcohol also appears to modify the risk for developing CCA in conjunction with exposures that increase nitrosamine exposure such as smoking and consumption of fermented food products [23]. Certain polymorphisms in the XRCC1 and OGG1 genes increased risk in combination with alcohol or smoking [26].

Though GSTM1 and GSTT1 polymorphisms alone were not risk factors, these genes modified the effect of other risk factors. Cases with the GSTM1 null polymorphism that also exhibited elevated *O. viverrini* antibody titers had over 23 times the odds of developing CCA over those with wild type GSTM1 [23]. The GSTT1 null polymorphism positively modified the effect of alcohol consumption, especially in ex-regular drinkers, who had over 27 times the odds of developing CCA [23]. Polymorphisms of the MTHFR gene interacted with dietary items, and certain polymorphisms were associated with increased risk of developing CCA in conjunction with consumption of raw fish dishes and fermented or processed meats [24, 27]. OGG1 in combination with polymorphisms of GSTM1 reduced risk of developing CCA, which the authors hypothesize is due to cell death before malignant transformation related to reduced DNA repair enzyme activity [30]. The CYP1A2*1A/*1A polymorphism was found to decrease the risk for developing CCA in men, and when male patients were further stratified by smoking status, the polymorphism reduced the risk of developing CCA by a factor of 14 in smokers [29]. Expression of the CYP1A2 gene may be related to exposures such as smoking and fermented and smoked meats that increase exposure to volatile nitrosamines [29].

### Meta-analyses

**Fig. 2:**
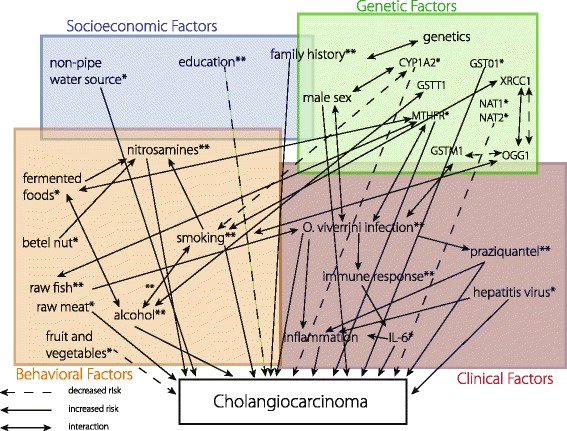
Relationships of risk factors for cholangiocarcinoma. * *P* < 0.05 based on individual study, ** *P* < 0.05 based on this meta-analysis

The results of random effects model meta-analyses performed for risk factors reported by two or more studies are reported in Table 5. Immune response to *O. viverrini* infection, measured by serum antibodies, significantly increased risk of developing CCA (*OR* = 6.09, 95% *CI*: 2.54–14.57, *P* <  0.0001). Increased alcohol consumption significantly increased risk of developing CCA, reported in five studies (*OR* = 2.61, 95% *CI*: 1.59–4.31, *P* = 0.002) [2, 22, 23, 25, 26]. Five studies reported the risk of smoking alone and indicated an increased risk of developing CCA from smoking (*OR* = 1.33, 95% *CI*: 1.00–1.78, *P* = 0.049). Meta-analysis of two studies reporting combined risk from alcohol consumption and smoking [23, 26] indicated markedly increased risk in people who both smoke and consume alcohol (*OR* = 11.1, 95% *CI*: 5.63–21.92, *P* <  0.0001). Meta-analysis of data from three studies [2, 23, 25] found that use of praziquantel was also significantly associated with increased risk of developing CCA (*OR* = 1.93, 95% *CI*: 1.2–3.1, *P* = 0.0065). Education was the only socioeconomic factor reported by two or more studies, and four studies [2, 22–24] indicated reduced risk of CCA associated with higher levels of education (*OR* = 0.46, 95% *CI*: 0.22–0.97, *P* = 0.04). A family history of cancer, reported by three studies [2, 22, 25], significantly increased risk for developing CCA (*OR* = 3.0, 95% *CI*: 1.79–5.04, *P* <  0.0001). Raw fish dishes, which are vectors of *O. viverrini*, significantly increased risk of developing CCA [22–25] (*OR* = 3.26, 95% *CI*: 1.58–6.71, *P* = 0.0014). High nitrate foods, inclusive of fermented or salted fish and meat and betel nut, also significantly increased risk for developing CCA (*OR* = 1.41, 95% *CI*: 1.05–1.91, *P* = 0.0241). Though fresh fruit and vegetable consumption significantly reduced risk for developing CCA in some studies, the pooled effects in meta-analysis were not significant. Betel nut, fermented fish, rice, and sticky rice were also examined during meta-analysis with no significant association with CCA development (Table 5). Figure 2 provides a causal diagram depicting significant individual and interacting factors and their associations with development of CCA.

**Table 5:** Random effects model meta-analyses of risk factors for cholangiocarcinoma

Risk factor	Total CCA Cases (Studies) Included in Analysis	Summary Effect Pooled *OR* (95% *CI*, *P*-value)	Heterogeneity I^2^ (*P*-value)
Behaviors			
Alcohol	682 (5) [2, 22, 23, 25, 26]	2.61 (1.59–4.31, 0.002)**	68% (0.01)
Smoking	772 (5) [2, 22, 23, 26, 29]	1.33 (1.00–1.78, 0.049)*	0% (0.56)
Alcohol x Smoking	348 (2) [23, 26]	11.1 (5.63–21.92, < 0.0001)***	0% (0.55)
Socioeconomics			
Education	559 (4) [2, 22–24]	0.46 (0.22–0.97, 0.04)*	51% (0.11)
Diet			
Fruit	682 (5) [2, 22–25]	0.8 (0.47–1.38, 0.431)	71% (0.01)
Vegetables	579 (4) [2, 23–25]	0.66 (0.38–1.13, 0.127)	48% (0.12)
Betel nut	355 (3) [22, 23, 25]	2.18 (0.62–7.63, 0.223)	54% (0.11)
Raw fish	574 (4) [22–25]	3.26 (1.58–6.71, 0.0014)**	53% (0.10)
Fermented fish	355 (3) [22, 23, 25]	1.18 (0.81–1.72, 0.401)	42% (0.11)
Fermented meats	471 (3) [23–25]	1.81 (0.96–3.39, 0.066)	17% (0.28)
High nitrate foods	682 (5) [2, 22–25]	1.41 (1.05–1.91, 0.024)*	46% (0.01)
Rice	232 (2) [22, 23]	0.88 (0.48–1.63, 0.688)	34% (0.22)
Sticky rice	232 (2) [22, 23]	1.69 (0.68–4.17, 0.258)	70% (0.07)
Genetics			
Family history	334 (3) [2, 22, 25]	3.0 (1.79–5.04, < 0.0001)***	0% (0.64)
Immune Response			
Anti-*O. viverrini* antibody	461 (4) [22, 23, 25, 38]	6.09 (2.54–14.57, < 0.0001)***	70% (0.03)
Treatment			
Praziquantel use	360 (3) [2, 23, 25]	1.93 (1.2–3.1, 0.0065)**	31% (0.25)
Other Infection			
Hepatitis B antigen	209 (2) [22, 38]	1.3 (0.59–2.85, 0.514)	0% (0.34)

Summary effect pooled odds ratio, 95% confidence interval (*CI*), and I^2^ were calculated using random effects models for risk factors reported by 2 or more studies in the systematic review. *P < 0.05, **P < 0.01, ***P < 0.001

### Quality assessment and limitations of included studies

Assessment of individual studies indicated that the majority of studies evaluated in this systematic review used a case control study design, so systematic recall bias must be considered since CCA is a cancer that develops over years of exposures. Patient reports may also be influenced by desirability bias based on what the patient perceives to be a desirable answer to report to physicians or researchers. Hospital or biological repository based sampling used in several studies also introduces bias, especially when control patients are selected from ill patients entering the hospital instead of healthy people in the community. Criteria for diagnosing CCA were variable between studies, with several allowing presumptive diagnosis via ultrasound examination instead of the gold standard by histology. Two studies used Japanese patients in the control group [31, 35] and compared with Thai CCA cases, so the differences in these populations should be considered. None of the studies provided sample size or power calculations in the design methods, and small sample size was a problem encountered with a number of studies. These differences among the studies contribute to heterogeneity, for which we have accounted by using random effects models for meta-analysis.

## Conclusions

To our knowledge, this is the first systematic review and meta-analysis to provide a comprehensive assessment of other risk factors for CCA described in the published literature and provides support for integrated interventions that consider the complexity of factor interrelations in the pathogenesis of CCA. Some of the described risk factors are primary and likely to be directly involved in the mechanism of pathogenesis of CCA, while others are secondary and may interact with or modify primary factors or represent a group of risk factors. Overall limitations of this systematic review to consider in light of these findings are the systematic recall bias and differences in measurements of exposures likely to be present across studies, as well as publication bias toward studies focused on *O. viverrini* as the primary, independent risk factor.

The combination of alcohol and smoking was the most significant risk factor, associated with over 11 times the odds of developing CCA in people who both smoke and consume alcohol, even greater than the risk from *O. viverrini* exposure as measured by antibody titers (Table 5). The risk of CCA related to smoking or alcohol consumption alone is also significantly increased and these are likely primary factors involved in carcinogenesis. A higher level of education in this meta-analysis was the only factor found to have a significant protective effect. This socioeconomic factor is an example of a risk factor that is not directly related to CCA pathogenesis, but represents the complex relationship of socioeconomic status with behaviors, stress, and exposure to other risk categories described in this analysis (Fig. 2).

Numerous dietary components were evaluated in the included studies. Our analysis found that raw fish dishes and high nitrate containing foods were significantly associated with risk of developing CCA. Several other food items were found to either increase or decrease risk in individual studies. Several traditional foods high in volatile nitrosamines, including fermented meat and fish dishes, processed meats, and betel nuts, were frequently investigated due to the known mechanism of nitrosamines in carcinogenesis and their dose-dependent interaction with *O. viverrini* infection [22, 23, 28]. Several of these food products significantly increased risk in individual studies (Table 2), and the random effects model for high nitrosamine foods, which compiled all reported foods of this category, was also significant (*P* = 0.02, Table 5). Smoking is also a major source of nitrosamine exposure, and in addition to diet, should be considered in the total nitrosamine load of individuals at risk (Fig. 2). Several studies identified fresh fruits and vegetables as significantly protective factors (Table 2). The vitamin C contained in many fruits and vegetables is thought to inhibit endogenous nitrosamine production associated with *O. viverrini* infection, which may contribute to the protective effect [23]. The result of the meta-analysis for the association of consumption of fruits and vegetables with CCA was not significant (Table 5), but the balance of the overall diet warrants further investigation in terms of nitrosamines, anti-oxidants, and other nutrients.

Numerous genetic factors were explored by the included studies, which indicate that genes involved in DNA repair, inflammation, and metabolism may increase or decrease risk of CCA (Table 3) and that certain genes may modify other exposures (Table 4). Family history of cancer was the only genetic-related risk factor that could be included in meta-analysis, and was highly significant (Table 5). Like education, family history is a risk factor that represents numerous others, and includes not only inherited genetic cancer risk, but also common environmental, socioeconomic, and psychosocial factors within families. A category of risk not described by studies included in this review is *O. viverrini* genetics, which varies geographically and appears to be related to the incidence of CCA in Thailand and Laos, though studies to compare genetic identities of *O. viverrini* in human patients have not yet been performed [7]. Praziquantel treatment was significantly associated with CCA in this meta-analysis. This likely represents another secondary risk factor that may be indicative of other relationships including *O. viverrini* infection intensity, human or parasite genetics, long-term endemicity of the parasite, misuse of the medication, or other effects of praziquantel, which have yet to be described in the research for CCA.

Given the relatively ineffective long-term nature of interventions focused at reducing *O. viverrini* infection prevalence and CCA incidence, especially in Northeast Thailand [12], this review suggests an evidence based approach that considers the socioeconomic, behavioral, clinical, and genetic risk factors identified (Fig. 2). Since risk factors such as alcohol consumption, smoking, and dietary habits are also associated with numerous other diseases, these findings indicate health behavior and education approaches tailored to the region’s specific social ecological characteristics [1, 19, 47]. This includes deeply embedded traditional eating and sanitation practices [48] and the disease burden now being faced with modernization [49] and a history of persistent poverty [50]. The social dynamics of food, as evidenced by food sharing social network analysis, demonstrates this important connection as households with higher overall social connectivity and sharing of fish dishes in Northeast Thailand had increased probability of *O. viverrini* infection [51].

Finally, a potentially important risk factor for CCA not examined by any epidemiological studies to date is exposure to toxic chemicals, of which pesticides and herbicides are particularly relevant in view of their increasing and largely un-regulated use by farmers in Thailand [1, 52]. Toxic chemical exposures are a known risk for CCA elsewhere, while the most widely used herbicide in Thailand, glyphosate [53], was recently classified by IARC as a probable carcinogen [54]. The case for including agrichemical exposures as a factor, glyphosate in particular, is especially compelling given that the same population most at risk for both *O. viverrini* infection and CCA – farmers – is that most exposed to glyphosate and other agrichemicals classified as carcinogens.

The CCA interventions to date have likely been less effective than anticipated due to their singular focus on *O. viverrini* infection, as well as their particular forms of approach to changing deeply held cultural behaviors in affected populations. In light of this, and of the findings in this review, we suggest a more integrated approach that more broadly accounts for the social, economic, and environmental determinants of CCA and may not only improve the outcomes of CCA interventions but positively impact public health more generally in the Lower Mekong region.

## Additional file

Additional file 1: Multilingual abstracts in the six official working languages of the United Nations. (PDF 758 kb)

## Abbreviations

CCA: Cholangiocarcinoma
IARC: International Agency for Research on Cancer
PRISMA: Preferred Reporting Items for Systematic Reviews and Meta-Analyses
WHO: World Health Organization
