# Supplementary material for: Thinking beyond Opisthorchis viverrini for risk of cholangiocarcinoma in the lower Mekong region: a systematic review and meta-analysis
Source: Infect Dis Poverty. 2018 May 17;7:44. doi: 10.1186/s40249-018-0434-3 (PMC5956617; doi:10.1186/s40249-018-0434-3)
Supplement: Supplementary file 1 — Multilingual abstracts in the six official working languages of the United Nations. (PDF 758 kb) [file 40249_2018_434_MOESM1_ESM.pdf]

Translation of the abstract into the six official working languages of the United Nations

التفكير لما هو أبعد من كون العدوى بطفيلي داء متأخر الخصية عامل الخطورة الوحيد للإصابة بسرطان الأقنية الصفراوية في منطقة حوض نهر ميكونج السفلي: مراجعة منهجية وتحليل إحصائي

قدمه: جينيفر أ. ستيل، وكارستن هـ.ريكتر، وبيير ايتشابارد، وباريتشات سينا، وفيرجينيا ستوت، و بايبون سيذيثاورن، وبروس أ ويلكوكس

الملخص

المعلومات الأساسية: يعد سرطان الأقنية الصفراوية من أورام القناة المرارية القاتلة ويحدث نتيجة العدوى بأحد أنواع المثقوبيات الكبدية لطفيل داء متأخر الخصية ، في منطقة حوض نهر ميكونج السفلي . وعلى الرغم من تركيز العديد من التدخلات الصحية العامة لتجنب العدوى بمتأخر الخصية كعامل مسبب لسرطان الأقنية الصفراوية ، لكن مازالت نسبة الإصابة بالمرض ترتفع في المنطقة . وبينما يشير ذلك إلى عدم كفاءة تلك التدخلات نتيجة للعديد من العوامل الإجتماعية والثقافية المعقدة، إلا أنه قد يشير لاحتمالية وجود عوامل خطورة أخرى أو تفاعلات مختلفة مع الطفيلي ذات أهمية في نشوء الأسباب المرضية لسرطان الأقنية الصفراوية. و تهدف هذه المراجعة المنهجية لتسليط الضوء على هذه العوامل وتحليلها موضوعيا باعتبارها مسببات أخرى لسرطان الأقنية الصفراوية بالإضافة إلى العدوى بمتأخر الخصية ليُسترشد بها في التدخلات الصحية المستقبلية.

الموضوع الرئيس: لقد قمنا بالبحث في خمسة قواعد بيانات الأبحاث العالمية وسبعة من قواعد بيانات الأبحاث التايلاندية بهدف إيجاد الدراسات المتعلقة بعوامل الخطورة التي قد تسبب الأصابة بسرطان الأقنية الصفراوية في منطقة حوض نهر ميكونج السفلي . وقد تم تقييم الدراسات المختارة لضمان الحيادية وعدم الإنحياز وضمان صحة العناصر المستوفاة كتعداد السكان وطرق تشخيص الإصابة بسرطان الأقنية الصفراوية والأساليب الإحصائية المستخدمة بالإضافة لجودة تصميم الدراسة ونوعها . وقد أظهرت الدراسات الثمانية عشر الأخيرة التي شملها البحث عدة عوامل قد تؤدي لخطر الإصابة بالمرض وتم تصنيفها كالآتي: عوامل تتعلق بالسلوكيات، والأوضاع الاقتصادية الاجتماعية، والنظام الغذائي، والعوامل الوراثية، والجنس، والاستجابة المناعية، وأمراض معدية أخرى، و بالإضافة إلى علاج العدوى بمتأخر الخصية. ذُكر سبعة عشر عاملاً من عوامل الخطورة في دراستين أو أكثر وتم تقييم هذه العوامل باستخدام نماذج الآثار العشوائية وذلك خلال إجراء التحليل التلوي. إذ يُشيرُ هذا التحليل التلوي أن تناول الكحوليات وتدخين السجائر في نفس الوقت (أو=11.1, 95% فاصل الثقة: 5.63–21.92, القيمة الاحتمالية<0.0001) يرتبطُ ارتباطاً وثيقاً بزيادة احتمالية خطر الإصابة بسرطان الأقنية الصفراوية بل قد يكون أكثر خطورة من التعرض لعدوى طفيل داء متأخر الخصية . وأوضحت نتائج التحليل أيضا أن التاريخ الأسري بالإصابة بمرض السرطان، وتناول أسماك الشبوطيات النيئة، واستهلاك المواد الغذائية الغنية بالنيترات، والعلاج بعقار البرازيكونتيل ترتبط بخطر مرتفع بشكل ملحوظ للإصابة. وقد يكون لهذه العوامل علاقات أكثر تعقيدا بالمضيف، أو الطفيلي، أو بكيفية نشوء الأسباب المرضية لسرطان الأقنية الصفراوية، وقد وُجِدَ أن هذه العوامل تتفاعل مع بعضها البعض حسب ما أشارت إليه أكثرمن دراسة.

الاستنتاجات: تشيرُ النتائج التي توصلنا إليها إلى تنوع عوامل الخطورة للإصابة بسرطان الأقنية الصفراوية بالإضافة إلى العدوى بطفيل متأخر الخصية والتي يجب أن تؤخذ بعين الاعتبار في تدخلات الصحة العامة المستقبلية للحد من انتشار الإصابة بالمرض في المناطق المتأثرة . وينبغي النظر على نحوٍ خاص في خطورة عامل تدخين السجائر وتناول الكحوليات، والأنظمة الغذائية المتبعة، والعوامل الاجتماعية الاقتصادية عند إعداد برامج التدخلات الصحية لتقليل نسبة الإصابة بسرطان الأقنية الصفراوية.

Translated from English version into Arabic by Salma Adel, proofread by Bashaier Allam, through

TRANSLATORS
WITHOUT BORDERS

湄公河下游地区麝猫后睾吸虫所致胆管癌的风险分析：系统综述和荟萃分析

Jennifer A. Steele, Carsten H. Richter, Pierre Echaubard, Parichat Saenna, Virginia Stout, Paiboon Sithithaworn, and Bruce A. Wilcox

摘要

引言：在湄公河下游地区，胆管癌(CCA)是一种致命的胆道系统恶性肿瘤，它与麝猫后睾吸虫的感染有关。许多公共卫生干预措施致力于减少与麝猫后睾吸虫的接触，但该地区 CCA 的发病率仍然很高。虽然这可能是由于复杂的社会和文化因素而导致的公共卫生干预效果不佳，但也进一步表明，其他危险因素或与寄生虫的相互作用在 CCA 的发病机制中也很重要。本系统综述旨在为 CCA 的风险因素提供全面的分析，以指导未来的综合干预措施。

主要内容：我们检索了5个国际和7个泰国研究数据库，以确定湄公河下游地区 CCA 相关风险因素的研究文献。对所选择的文献就研究设计、人群、CCA 诊断方法和统计方法进行风险偏倚和质量评估。最后纳入的18项研究文献报告了许多风险因素，包括行为、社会经济情况、饮食、遗传、性别、免疫反应、其他感染和 CCA 治疗。17个风险因素是由两个或两个以上的研究报道并在荟萃分析中进行随机效应模型评估。荟萃分析表明，酒精和吸烟的组合[OR = 11.1, 95%置信区间(CI): 5.63–21.92, $P < 0.0001$]是最重要的风险因素，与 CCA 风险增加显著相关，比暴露于麝猫后睾吸虫的风险更高。癌症家族史、食用生鲤鱼、食用高硝酸盐食物和吡喹酮治疗与风险增高显著相关。这些风险因素可能与宿主、寄生虫或 CCA 的发病机制有复杂关系，在不止一项研究中发现，许多风险因素与其他风险因素均存在交互作用。

结论：本研究结果表明，除了麝猫后睾吸虫感染外，在未来的公共卫生干预措施中，还应处理各种复杂的危险因素，以降低受影响地区的 CCA 发病率。在制定降低 CCA 的干预计划时，应该考虑吸烟和饮酒、饮食模式和社会经济因素。

Translated from English version into Chinese by Translated by Xin-Yu Feng, edited by Pin Yang

CHINESE CENTER FOR DISEASE CONTROL AND PREVENTION
NATIONAL INSTITUTE OF PARASITIC DISEASES

Le risque de cholangiocarcinome dans la région du bas-Mékong, au-delà d'*Opisthorchis viverrini* : revue systématique et méta-analyse

Jennifer A. Steele, Carsten H. Richter, Pierre Echaubard, Parichat Saenna, Virginia Stout, Paiboon Sithithaworn et Bruce A. Wilcox

Résumé

Contexte: Le cholangiocarcinome est un cancer fatal des voies biliaires associé à l'infestation par la douve *Opisthorchis viverrini* dans la région du bas-Mékong. Bien que de nombreuses interventions de santé publique aient été réalisées dans le but de réduire l'exposition à *O. viverrini*, l'incidence du cholangiocarcinome reste élevée dans la région. Cela peut témoigner de l'inefficacité de ces interventions, en raison de facteurs sociaux et culturels complexes. On peut cependant y voir aussi l'indication que d'autres facteurs de risque ou interactions avec le parasite entrent en ligne de compte dans la pathogenèse du cholangiocarcinome. La présente revue systématique a pour but de donner une analyse complète des facteurs de risque de cholangiocarcinome décrits en plus d'*O. viverrini*, afin d'orienter des interventions intégrées futures.

Discussion: Nous avons effectué une recherche dans cinq bases de données de recherche internationales et sept bases de données thaïlandaises afin d'identifier des études concernant les facteurs de risque de cholangiocarcinome dans la région du bas-Mékong. Le risque de biais des études sélectionnées et leur qualité (plan d'étude, population, méthodes de diagnostic du cholangiocarcinome et méthodes statistiques) ont été évalués. Les dix-huit études finalement incluses font état de nombreux facteurs de risque, qui ont été regroupés en catégories : comportements, facteurs socioéconomiques,

génétique, sexe, réponse immunitaire, autres infections et parasitoses et traitement contre *O. viverrini*. Dix-sept facteurs de risque ont été rapportés par deux ou plusieurs études et ont été évalués à l'aide de modèles à effets aléatoires au cours de la méta-analyse. La méta-analyse indique que la combinaison de la consommation d'alcool et de tabac (*OR* = 11,1, *IC* à 95 %de 5,63 à 21,92, $P < 0{,}0001$) est très significativement associée au risque de cholangiocarcinome et qu'il s'agit d'un facteur de risque encore plus important que l'exposition à *O. viverrini*. Cette analyse suggère également que les antécédents familiaux de cancer, la consommation de poissons cyprinoïdes crus, la consommation d'aliments à teneur élevée en nitrates et les traitements par le praziquantel sont associés à une élévation significative du risque. Ces facteurs de risque peuvent avoir des relations complexes avec l'hôte, le parasite ou la pathogenèse du cholangiocarcinome et il est apparu que plusieurs d'entre eux interagissaient les uns avec les autres dans une ou plusieurs études.

Conclusions: Nos résultats suggèrent que des facteurs de risque divers et complexe s'ajoutent à l'infestation par *O. viverrini*. Les futures intervention de santé publique devront également s'intéresser à ces facteurs afin de réduire l'incidence du cholangiocarcinome dans les régions affectées. L'élaboration des programmes d'intervention devra viser, en particulier, la consommation d'alcool et de tabac, les habitudes alimentaires et les facteurs socioéconomiques.

Translated from English version into French by Suzanne Assenat, proofread by WillSquire, through

TRANSLATORS WITHOUT BORDERS

Системный обзор и мета-анализ риска заболевания холангиокарциномой, как следствия заражения *Opisthorchis viverrini* в нижней части бассейна реки Меконг

Дженнифер А. Стил, Карстен Х. Рихтер, Пьер Эшаубард, Парихат Саенна, Вирджиния Стаут, Пайбун Сититаворн и Брюс А. Уилкокс

Аннотация

Контекст: Холангиокарцинома (CCA) - смертельный вид рака желчных протоков, связанный с инфицированием беличьей двуусткой, *Opisthorchis viverrini*, которая встречается в нижнем бассейне реки Меконг. Многочисленные мероприятия в области здравоохранения были направлены на снижение инвазии *O. viverrini*, но распростаненность заболевания в регионе остается высокой. Это может означать и неэффективность мероприятий в сфере здравоохранения по социальным и культурным причинам, и также может означать, что важными факторами в патогенезе холангиокарциномы играют взаимодействие с паразитом и иные факторы риска. Цель этого системного обзора - предоставить комплексный анализ обозначенных факторов риска в заболевании холангиокарциномой, вызванной *O. viverrini* , в целях принятия комплексных мер в будущем .

Главная часть: Мы изучили пять международных и семь тайландских исследовательских баз данных для обзора работ, посвященных факторам риска заболевания холангиокарциномой в районе нижнего бассейна реки Меконг. Отдельные исследования оценивались на предмет риска системных ошибок и качества с точки зрения структуры исследования, численности населения, методов диагностики холангиокарциномы (CCA) и статистических методов. Последние восемнадцать исследований сообщают о множественных рисках, объединенных по параметрам поведения, социоэкономических и генетических факторов, диеты, пола, реакции иммунной системы, других инфекций и лечения *O. viverrini.* Более двух исследований сообщают о семнадцати факторах риска,

которые были оценены по выборочным моделям в ходе мета-анализа. Данный мета-анализ указывает на то, что повышенный риск заболевания холангиокарциномой в существенной мере связан с сочетанием алкоголя и курения (*ИЛИ*=11.1, 95% *CI*: 5.63–21.92, $P < 0.0001$), и это даже больший фактор риска, чем инвазия *O. viverrini* . Данный анализ также предполагает, что генетическая предрасположенность к раку, потребление сырой рыбы семейства карповых и пищи с высоким содержанием нитратов, а также лечение празикантелем являются факторами риска. Эти факторы риска могут быть по разному связаны с носителем, паразитом или влиять на патогенез холангиокарциномы, и в нескольких исследованиях обнаружено взаимодействие этих факторов риска.
Заключение: Наши результаты свидетельствуют о том, что для снижения заболеваемости холангиокарциномой в пораженных регионах необходимы мероприятия по борьбе с комплексом различных факторов риска наряду с инвазией *O. viverrini*. При разработке программы практических мер для снижения заболевания холангиокарциномой должны быть приняты во внимание в частности, курение и злоупотребление алкоголем, режим питания и социоэкономические факторы.

Translated from English version into Russian by Lisazaveta Frolova, proofread by Tatiana Kary, through

TRANSLATORS WITHOUT BORDERS

Pensando más allá de la Duela hepática china *Opisthorchis viverrini por el riesgo de* colangiocarcinoma en la región baja de Mekong: una revisión sistemática y un meta- análisis

Jennifer A. Steele, Carsten H. Richter, Pierre Echaubard, Parichat Saenna, Virginia Stout, Paiboon Sithithaworn, y Bruce A. Wilcox

Abstracto

Antecedentes: Colangiocarcinoma (CCA) es un cáncer fatal de las vías biliares asociado con una infección producida por la fasciola hepática, *Opisthorchis viverrini*, en la región baja de Mekong. Numerosas intervenciones de salud pública se han enfocado en reducir la exposición a la *O. viverrini*, pero la incidencia del CCA en la región permanece alta. Mientras que esto puede indicar la ineficacia de las intervenciones de salud pública debida a factores sociales y culturales, puede indicar más profundamente otros factores de riesgo o interacciones con el parásito que son importantes en la patogénesis del CCA. Esta revisión sistemática busca proveer un análisis comprehensivo de los factores de riesgo descritos del CCA sumado al *O. viverrini* para guiar futuras intervenciones integrativas.
Main Body: Buscamos cinco bases de datos internacionales y siete Thai para identificar estudios que sean relevantes a factores de riesgo del CCA en la región baja de Mekong. Estudios seleccionados fueron evaluados por riesgo de preferencia y calidad en términos de diseño de estudio, población, métodos de diagnóstico de CCA y métodos estadísticos. Los últimos dieciocho incluyeron estudios que reportaron varios factores de riesgo los cuales fueron agrupados en conductas, nivel socioeconómico, dieta, genética, género, respuesta inmunitaria, otras infecciones, tratamiento para *O. viverrini.* Diecisiete factores de riesgo fueron reportados por uno o más estudios y fueron evaluados con modelos de efectos aleatorios durante el meta-análisis. Este meta-análisis indica que la combinación de alcohol con fumar (*OR*=11.1, 95% *CI*: 5.63–21.92, $P < 0.0001$) está significativamente más asociada con el incremento de riesgo de CCA y es un evento de mayor riesgo que la exposición al *O. viverrini*. Este análisis también sugiere el historial familiar

de cáncer, el consumo de peces ciprínidos crudos, el consume de alimentos con alto nitrato, y el tratamiento con praziquantel están asociados a un riesgo significativamente incrementado. Estos factores de riesgo pueden tener relaciones complejas con el portador, el parásito la patogénesis del CCA, y se encontró que muchos de estos factores de riesgo interactúan entre sí en varios estudios.

Conclusiones: Nuestros hallazgos sugieren que una variedad compleja de factores de riesgo adicional a la infección *O. viverrini* deberían ser tratados en intervenciones futuras de salud pública para reducir el CCA en regiones infectadas. En particular, fumar y el uso de alcohol, patrones de dieta, y factores socioeconómicos deben ser considerados cuando se desarrollen programas de intervención para reducír el CCA.

Translated from English version into Spanish by Iñaki Vega Bayo, proofread by Alexis ponce, through

TRANSLATORS
WITHOUT BORDERS
